# New Professorship

**Published:** 1852-11

**Authors:** 


*New Professorship.*—We understand that John A. Swett, M. D., of the N. Y. Hospital, has been appointed Professor of Clinical Medicine, in the College of Physician and Surgeons of this city, and will deliver a course of lectures on this department during the approaching session.

We rejoice at this addition to the curriculum of this college, evincing as it does a just estimate of the indispensable necessity of thorough clinical instruction, in every medical school worthy of the name. Dr. Swett is a learned and able teacher, a successful practitioner, and has a reputation at home and abroad which will render him a real acquisition to the faculty of the college. —*Medical Gazette.*

